# Correlation between systemic inflammatory response index and post-stroke epilepsy based on multiple logistic regression analysis

**DOI:** 10.3389/fneur.2025.1640796

**Published:** 2025-10-03

**Authors:** Yong Mei Hu, Hua Huang, Yu Ting Chen, Wen Jin Wang, Bai Hui Zhang

**Affiliations:** ^1^College of Life and Sciences, Chengdu University of Traditional Chinese Medicine, Chengdu, China; ^2^Department of Neurology, Dazhou Central Hospital, Dazhou, China

**Keywords:** stroke, epilepsy, systemic inflammatory response index, inflammation, risk factors, landmark

## Abstract

**Background:**

Post-stroke epilepsy (PSE) is an important neurological complication affecting the prognosis of stroke patients. Recent studies have found that the systemic inflammatory response index (SIRI) is a new inflammatory marker, and its mechanism of association with PSE is not yet clear. The purpose of this study was to investigate the correlation between SIRI level and the occurrence of PSE.

**Methods:**

The study retrospectively included 226 stroke patients admitted from July 2021 to October 2024. According to the occurrence of epilepsy, they were divided into PSE group (*n* = 57) and non-PSE group (*n* = 169). Multivariate Logistic regression analysis was used to evaluate the correlation strength between SIRI and PSE, and the Restricted Cubic Spline (RCS) model was used to explore the strong nonlinear relationship between SIRI and PSE. At the same time, the stratified analysis method was used to deeply explore the basic disease status and correct the following variables: (1) Demographic characteristics: social demographic indicators such as age, gender, BMI and education level were included; (2) lifestyle factors: including smoking status and drinking habits; (3) complications: clinical diagnosed diseases such as hypertension, diabetes, coronary heart disease and chronic obstructive pulmonary disease; (4) laboratory test parameters: including neutrophils, lymphocytes, monocytes and other blood cell classification counts, as well as biochemical indicators such as platelets, hemoglobin, total protein and total cholesterol.

**Results:**

The baseline SIRI level in the PSE group was significantly higher than that in the non-PSE group (3.43 ± 2.74 vs. 1.79 ± 1.40, *p* < 0.001). Stratified analysis showed that there was a significant interaction between SIRI and PSE in the subgroup with underlying diseases (*p* < 0.001). The RCS analysis also suggested that there was a nonlinear positive correlation between SIRI and PSE risk (*p* = 0.382), and the risk inflection point appeared when SIRI = 1.36.

**Conclusion:**

This study shows that elevated SIRI is associated with the occurrence of PSE, especially in stroke patients with underlying diseases. The results of this study provide a new reference of inflammatory biomarkers for early warning and hierarchical management of PSE.

## 1 Introduction

Stroke refers to acute neurological deficits caused by cerebrovascular accidents (such as cerebral infarction or cerebral hemorrhage). The incidence of stroke increases significantly with age and is common in the elderly (1, 2). According to epidemiological data, 10.2% of all deaths worldwide in 2016 were attributable to stroke, making it the second leading cause of death worldwide (3). It is worth noting that complications often occur after the acute phase of stroke. Among them, epilepsy is a common complication after stroke, with an incidence of 2 -15%. For newly diagnosed epilepsy patients over 65 years old, the main cause is stroke, namely post-stroke epilepsy (4, 5). Epileptic seizures are mainly caused by mechanisms such as ischemia and hypoxia, cell death and inflammatory response in brain tissue after stroke (6). In recent years, the mechanism of inflammatory response in the development of PSE has been the focus of attention at home and abroad (7, 8). Systemic inflammation response index (SIRI), as a new non-specific inflammatory marker, integrates neutrophils, monocytes and lymphocytes, reflects systemic inflammation, and is cost-effective and easy to detect (9). A recently emerging inflammatory index may be related to PSE. Based on the above research background, this study plans to explore the correlation between SIRI and PSE, aiming to provide a new theoretical basis for the early prevention and treatment of stroke complications.

## 2 Data and methods

### 2.1 Data

#### 2.1.1 Study population

**Figure 1:**
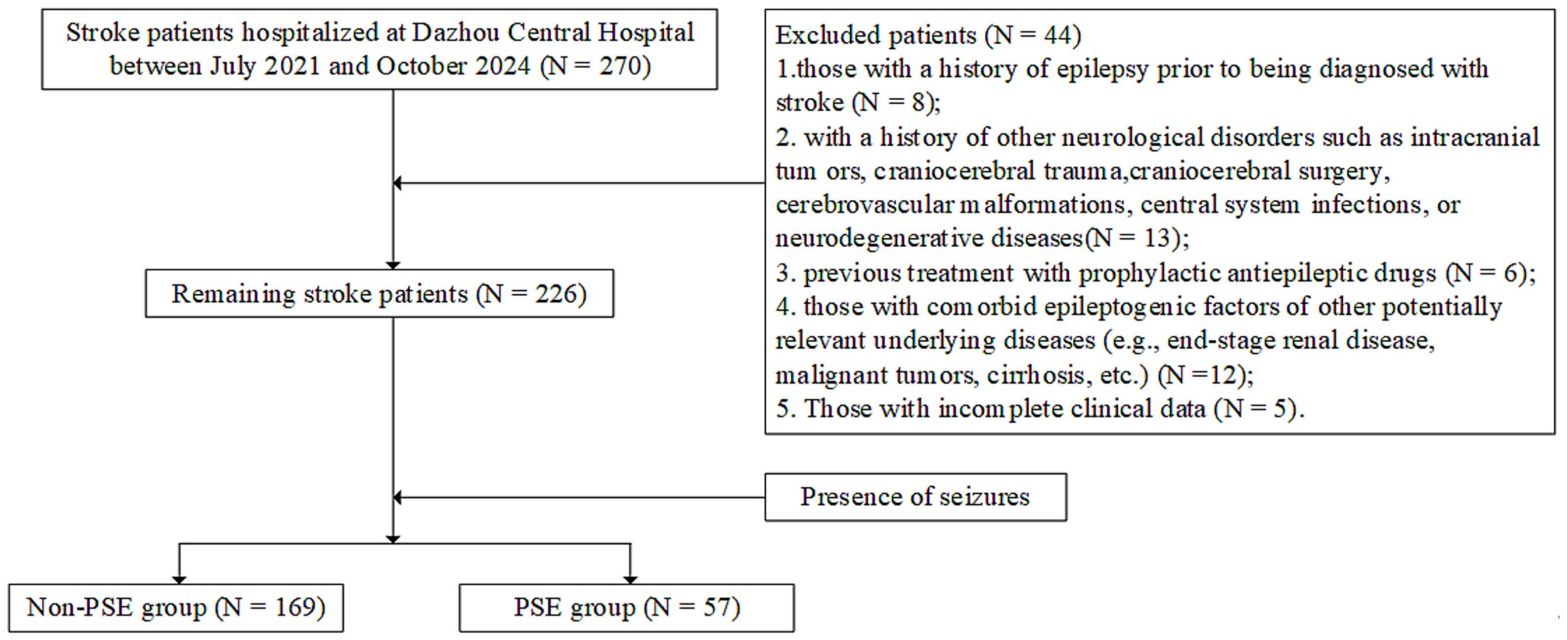
The research process.

This retrospective study included 270 consecutive stroke patients admitted to Dazhou Central Hospital from July 2021 to October 2024. The inclusion criteria of patients were as follows: (1) The diagnosis of stroke should meet the diagnostic criteria of stroke revised by the consensus expert committee of the cerebrovascular group of the Chinese Society of Neurology (10), and confirmed by computed tomography (CT) or magnetic resonance imaging (MRI) of the head; (2) the first stroke occurred in our hospital; (3) there was no history of epileptic seizures in the past, and the symptoms of epileptic seizures occurred after stroke, which met the diagnostic criteria of ILAE in 2017 (11). (4) Age≥18 years old. Exclusion criteria: (1) patients with a history of epilepsy before stroke; (2) accompanied by traumatic brain injury, brain surgery, cerebrovascular malformations, central nervous system infection or neurodegenerative diseases and other history of nervous system diseases; (3) those who had previously taken preventive antiepileptic drugs; (4) patients with other underlying diseases (such as end-stage renal disease, malignant tumor, cirrhosis, etc.) and incomplete clinical data. Among 226 eligible stroke patients, patients were divided into PSE group (*n* = 57) and non-PSE group (*n* = 169) according to whether epilepsy occurred after stroke. The patient selection process is shown in Figure 1. The study was approved by the Ethics Committee of Dazhou Central Hospital.

### 2.2 Methods

#### 2.2.1 Clinical data collection

In this study, the complete clinical baseline data and laboratory test data of the subjects were collected through the hospital electronic medical record system. Based on previous studies, the risk of recurrence in patients with status epilepticus after stroke is closely related to a variety of clinical factors, including the severity of stroke (such as high NIHSS score), stroke site (such as cortical involvement), stroke type (such as cerebral hemorrhage or cardiogenic cerebral embolism), early epileptic seizures and previous history of patients (including drinking history, diabetes, hypertension and other basic diseases), and some research results have been achieved (12). The study of Takase et al. (13) showed that patients with PSE after cardiogenic cerebral infarction often had higher blood glucose levels. The reason may be that hyperglycemia can aggravate nerve cell damage through a variety of harmful pathways including oxidative stress. Therefore, this study adjusted socio-demographic variables (age, gender, BMI, education level), laboratory data (total cholesterol, triglyceride, low-density lipoprotein cholesterol, high-density lipoprotein cholesterol, etc.), lifestyle factors (drinking history, smoking history) and history of underlying diseases (including hypertension, diabetes, coronary heart disease and chronic obstructive pulmonary disease) as potential confounding factors. The specific collected indicators include: (1) Demographic characteristics: age, gender, education level and BMI; (2) lifestyle: including smoking history (defined as an average of at least one cigarette per day over the past year) and drinking history (defined as daily intake≥100 mL and alcohol content≥50% for at least 1 year); (3) complications: clinically diagnosed chronic diseases such as hypertension, diabetes, coronary heart disease and chronic obstructive pulmonary disease; (4) laboratory test data within 24 h after the first admission: (1) Blood cell parameters: neutrophil count (N), monocyte count (M), lymphocyte count (L), platelet count (PLT) and hemoglobin (Hb); (2) lipid metabolism indexes: total cholesterol (TC), triglyceride (TG), high density lipoprotein cholesterol (HDL-C), low density lipoprotein cholesterol (LDL-C); (3) biochemical indicators: total protein (TP) and serum electrolytes (sodium, potassium, calcium, chloride ion concentration). Venous blood samples were collected and analyzed by the hospital laboratory using the following methods. The diagnosis of hypertension, diabetes, coronary heart disease and chronic obstructive pulmonary disease was confirmed by the relevant specialists.

#### 2.2.2 Serum index levels

The levels of peripheral blood neutrophils, monocytes, lymphocytes and platelets, hemoglobin and other indicators were measured. Fasting venous blood 3 mL was extracted from patients within 24 h of admission, and the samples were mixed upside down for 8 times and then tested directly on the machine. The detection instrument was Beckman Xisen Meikang SXSmex XN-20 automatic blood analyzer. The levels of total cholesterol, triglyceride, high-density lipoprotein cholesterol, low-density lipoprotein cholesterol, total protein, hemoglobin and electrolytes were measured. Fasting venous blood 3 mL was collected within 24 h after admission. The serum was separated by centrifuge at 3,000 r/min (centrifugal radius 15 cm) for 10 min. The detection instrument was Beckman BECKMAN COULTER, Au-5800 automatic biochemical analyzer. The above operations were performed by the laboratory physician of our hospital.

#### 2.2.3 PSE diagnostic criteria

Post-stroke epilepsy refers to patients who have no history of epilepsy before stroke, have seizures within a certain period of time after stroke, meet the diagnostic criteria of epilepsy of the international league against epilepsy (ILAE), and exclude other brain lesions and systemic diseases. The epileptic discharge of EEG is consistent with the location of stroke (14). PSE diagnosis was assessed by two experienced neurologists.

#### 2.2.4 SIRI index calculation

SIRI is defined as follows: SIRI = M × N/L, where M, N and L represent the counts of monocytes, neutrophils and lymphocytes, respectively (15).

### 2.3 Statistical analysis

Excel 2020 is used for data entry, and double independent entry and error verification are performed to ensure data accuracy. Subsequent statistical analysis was performed in R 4.1.3 environment. Among them, the categorical variable is expressed as frequency (%), and the continuous variable is expressed as mean±standard deviation (IQR). Normality was evaluated by Kolmogorov–Smirnov test. Continuous variables between groups were compared using the t test or the Mann–Whitney U test (if applicable). Chi-square test was used to analyze categorical variables. In this study, we use logistic regression analysis because it facilitates the treatment of binary outcome variables while adjusting for potential confounding factors. Restricted cubic spline (RCS) curve can more accurately describe the relationship between continuous variables and outcome risk. The SIRI was divided into quartiles (Q1-Q4), and the Q1 group was used as a logistic regression reference group. Model 1 is not adjusted for covariates. Model 2 was adjusted according to gender, age and BMI. In Model 3, we adjusted all covariates (age, gender, education level, BMI, smoking, drinking, diabetes, hypertension, coronary heart disease, chronic obstructive pulmonary disease, neutrophils, monocytes, lymphocytes, platelets, hemoglobin, total protein, total cholesterol, triglycerides, high-density lipoprotein cholesterol, low-density lipoprotein cholesterol and serum sodium, potassium, calcium, chlorine). We also conducted a subgroup analysis of people with or without underlying diseases to assess the differences between them. In addition, we use RCS to explore the linear relationship between SIRI and PES risk. *p* < 0.05 was considered statistically significant.

## 3 Results

### 3.1 Baseline characteristics of overall patients

The analysis included 226 stroke patients. The study group included 122 males (53.98%) and 104 females (46.02%). The average age of the study population was 68.07 ± 9.18 years. The average BMI was 24.01 ± 3.22. There were 190 cases (84.07%) with junior high school education and below and 36 cases (15.93%) with high school education and above. There were 139 non-smokers (61.50%) and 87 smokers (38.50%). There were 156 (69.03%) patients who did not drink alcohol and 70 (30.97%) patients who drank alcohol. There were 65 patients (28.76%) without underlying diseases and 161 patients (71.24%) with underlying diseases. Stroke without epilepsy 169 (74.78%) and post-stroke epilepsy 57 (25.22%). Most of the study participants did not smoke or drink. The proportion of studies on diabetes, coronary heart disease, and chronic obstructive pulmonary disease was also lower than that of previous medical history, while the proportion of studies on hypertension was the opposite. Table 1 lists the characteristics of patients in the study cohort.

**Table 1:** Overall patient baseline (*N* = 226).

Variant	
Sex	
Male	122 (53.98%)
Female	104 (46.02%)
Age	68.07 ± 9.18
Height	160.28 ± 9.21
Weight	61.86 ± 10.62
BMI	24.01 ± 3.22
Education attainment	
Junior high school and below	190 (84.07%)
High school and above	36 (15.93%)
Smoking	
No	139 (61.50%)
Yes	87 (38.50%)
Drinking Wine	
No	156 (69.03%)
Yes	70 (30.97%)
Underlying Disease	
No	65 (28.76%)
Yes	161 (71.24%)
High Blood Pressure	
No	83 (36.73%)
Yes	143 (63.27%)
Diabetes	
No	174 (76.99%)
Yes	52 (23.01%)
Coronary Heart Disease	
No	207 (91.59%)
Yes	19 (8.41%)
Chronic Obstructive Pulmonary Disease	
No	223 (98.67%)
Yes	3 (1.33%)
Post-stroke Epilepsy	
No	169 (74.78%)
Yes	57 (25.22%)

### 3.2 Descriptive statistics of general population examination results

Descriptive analysis was performed on the laboratory examination results of stroke patients after admission. The median platelet count was 176.29 ± 60.26 × 109/L, and the median neutrophil count was 5.01 ± 2.38 × 109/L. The median lymphocyte count was 1.52 ± 0.6 ×109/L. The median monocyte count was 0.56 ± 0.28 × 109/L The median total cholesterol was 4.53 ± 1.09 mmol/L. The median of high density lipoprotein cholesterol was 1.31 ± 0.44 mmol/L. The median level of low density lipoprotein cholesterol was 2.61 ± 0.88 mmol/L. The median of triglyceride was 1.62 ± 0.85 mmol/L. The median hemoglobin was 130.13 ± 17.69 mmol/L. The median of total protein was 66.17 ± 6.68. The median of serum potassium was 3.82 ± 0.48. The median of serum sodium was 140.15 ± 4.29. The median of serum calcium was 2.27 ± 0.15. The median of serum chlorine was 104.02 ± 10.44. Laboratory indicators Table 2 lists the detailed results.

**Table 2:** Laboratory findings of the patients.

Variant	Min	P25	P50	Mean ± sd	P75	Max
Total cholesterol	1.10	3.84	4.46	4.53 ± 1.09	5.09	8.21
High density lipoprotein	0.41	1.05	1.24	1.31 ± 0.44	1.49	4.54
Low density lipoprotein	0.59	1.99	2.56	2.61 ± 0.88	3.22	5.42
Triglyceride	0.51	1.08	1.42	1.62 ± 0.85	1.91	5.83
Hemoglobin	64.00	119.00	132.00	130.13 ± 17.69	142.00	182.00
Total protein	32.30	62.06	65.75	66.17 ± 6.68	70.09	90.50
Potassium	1.04	3.57	3.85	3.82 ± 0.48	4.10	5.17
Sodium	106.60	138.00	140.80	140.15 ± 4.29	142.90	150.00
Calcium	1.47	2.20	2.29	2.27 ± 0.15	2.36	2.63
Chlorine	1.30	102.80	105.70	104.02 ± 10.44	107.10	118.20
Monocyte	0.06	0.39	0.52	0.56 ± 0.28	0.64	2.30
Lymphocyte	0.43	1.11	1.44	1.52 ± 0.60	1.85	3.67
Neutrophil	1.55	3.46	4.38	5.01 ± 2.38	6.04	15.40
Platelet count	29.00	135.25	172.50	176.29 ± 60.26	212.50	445.00

TC, total cholesterol; LDL-C, low density lipoprotein cholesterol; HDL-C, high density lipoprotein cholesterol; TG, triglyceride; HB, hemoglobin; TP, total protein; serum K+, serum potassium; serum Cl^−^, serum chlorine; serum Ca^2+^, serum calcium; serum Na+, serum sodium; M, monocyte count; L, lymphocyte count; N, neutrophil count; PLT, platelets.

### 3.3 Differences between the two groups

This study finally included all stroke patients with complete data (Figure 1). Among them, 169 were non-PSE patients and 57 were PSE patients. In the PSE group, women accounted for 49.12% and men accounted for 50.88%. SIRI was 3.43 ± 2.74. The differences in total cholesterol, triglycerides, neutrophils and SIRI between the two groups were statistically significant (*p* < 0.001), while the differences in age, gender, education level, BMI, smoking, alcohol consumption, underlying diseases, HDL cholesterol, LDL cholesterol, hemoglobin, total protein, monocytes, lymphocytes, platelets, and serum electrolytes (sodium, potassium, calcium, chloride) were not statistically significant (*p* > 0.001), as shown in Table 3.

**Table 3:** Baseline characteristics of the two groups.

Variant	Stroke (*n* = 169)	Post-stroke epilepsy (*n* = 57)	*p*
Sex			0.696
Male	93 (55.03%)	29 (50.88%)	
Female	76 (44.97%)	28 (49.12%)	
Age	66.53 ± 9.20	72.61 ± 7.51	<0.001
BMI	24.07 ± 3.45	23.86 ± 2.40	0.614
Education attainment			0.552
Junior high school and below	144 (85.21%)	46 (80.70%)	
High school and above	25 (14.79%)	11 (19.30%)	
Smoking			0.650
No	102 (60.36%)	37 (64.91%)	
Yes	67 (39.64%)	20 (35.09%)	
Drinking wine			0.346
No	120 (71.01%)	36 (63.16%)	
Yes	49 (28.99%)	21 (36.84%)	
Underlying disease			0.020
No	56 (33.14%)	9 (15.79%)	
Yes	113 (66.86%)	48 (84.21%)	
Total cholesterol	4.31 ± 0.94	5.18 ± 1.23	<0.001
High density lipoprotein	1.32 ± 0.47	1.29 ± 0.35	0.584
Low density lipoprotein	2.64 ± 0.90	2.51 ± 0.83	0.299
Triglyceride	1.45 ± 0.64	2.15 ± 1.13	<0.001
Hemoglobin	130.88 ± 17.96	127.91 ± 16.84	0.261
Total protein	66.85 ± 6.46	64.15 ± 6.95	0.011
Potassium	3.81 ± 0.50	3.85 ± 0.43	0.589
Sodium	140.42 ± 4.37	139.34 ± 3.97	0.087
Calcium	2.28 ± 0.15	2.23 ± 0.13	0.038
Chlorine	103.85 ± 11.81	104.52 ± 4.37	0.534
Monocyte	0.52 ± 0.23	0.66 ± 0.37	0.010
Lymphocyte	1.57 ± 0.62	1.39 ± 0.52	0.033
Neutrophil	4.59 ± 2.03	6.25 ± 2.90	<0.001
Platelet count	173.53 ± 59.72	184.49 ± 61.62	0.245
SIRI	1.79 ± 1.40	3.43 ± 2.74	<0.001

PSE, post-stroke epilepsy; BMI, body mass index; TC, total cholesterol; TG, triglyceride; LDL-C, low density lipoprotein cholesterol; HDL-C, high density lipoprotein cholesterol; L, lymphocyte count; M, monocyte count; N, neutrophil count; PLT, platelet; HB, hemoglobin; TP, total protein; serum K+, serum potassium; serum Cl^−^, serum chlorine; serum Ca^2+^, serum calciu; serum Na^+^, serum sodium; SIRI, systemic inflammatory response index.

### 3.4 The relationship between SIRI and post-stroke epilepsy

In the overall population, univariate logistic regression results showed that SIRI was positively correlated with PSE (OR: 1.49, 95% CI: 1.27–1.76). After adjusting for gender, age and BMI in model 2, this relationship still existed (OR: 1.46, 95% CI: 1.23–1.73). In model 3, after adjusting all covariates, SIRI was still significantly associated with the incidence of PSE (OR: 1.99, 95% CI: 1.44–2.75, *p* < 0.001). With Q1 as a reference, when SIRI was divided into quartiles, the OR value of Q4 was significantly higher than that of Q1 (OR: 5.58, 95% CI: 2.16–14.40, *p* < 0.001). After adjusting for gender, age, and BMI in Model 2, the OR value of the highest quartile of SIRI Q4 was still higher than that of Q1 (OR: 4.94, 95% CI: 1.83–13.36, *p* < 0.001). After full correction of all covariates, the risk of disease in patients with the highest quartile of SIRI was still significantly higher than that in patients with the lowest quartile (OR: 13.84, 95% CI: 3.19–60.03, *p* < 0.001). The detailed results are shown in Table 4.

**Table 4:** Relationship between SIRI level and prevalence of PSE by logistic regression analysis.

	Model 1		Model 2		Model 3	
	OR (95%CI)	*p*-value	OR (95%CI)	*p*-value	OR (95%CI)	*p*-value
SIRI	1.49(1.27 ~ 1.76)	<0.001	1.46 (1.23 ~ 1.73)	<0.001	1.99 (1.44 ~ 2.75)	<0.001
SIRI (quartile)						
Q1 (<0.98)	Reference		Reference		Reference	
Q2 (0.98–1.36)	1.71(0.61 ~ 4.78)	0.308	1.88 (0.63 ~ 5.63)	0.260	1.84(0.47 ~ 7.10)	0.379
Q3 (1.36–2.79)	2.44(0.90 ~ 6.61)	0.080	2.54 (0.90 ~ 7.16)	0.079	2.04(0.52 ~ 7.98)	0.307
Q4 (> = 2.79)	5.58(2.16 ~ 14.40)	<0.001	4.94 (1.83 ~ 13.36)	0.002	13.84(3.19 ~ 60.03)	<0.001
*p* for trend	<0.001		<0.001		<0.001	

Model 1: No covariates were adjusted. Model 2: Adjusted for age, gender, BMI. Model 3: Adjusted for age, gender, BMI, education level, smoking status, drinking; coronary heart disease, chronic obstructive pulmonary disease, diabetes, hypertension, TC, TG, LDL-C, HDL-C, L, M, N, PLT, HB, TP, Serum K^+^, Serum Cl^−^, Serum Ca^2+^, Serum Na^+^. 95% CI, 95% confidence interval.

### 3.5 Subgroup analysis

To verify the stability of the relationship between SIRI and post-stroke epilepsy in different subgroups, subgroup analyses were performed based on Model 3. The results are shown in (Table 5). There was a significant interaction of underlying disease between SIRI and post-stroke epilepsy (*p* < 0.001), indicating that the relationship between elevated SIRI and post-stroke epilepsy was more pronounced in the stroke population with underlying disease. However, the interaction was not significant in the stroke population without underlying disease (*p* > 0.001 for interaction).

**Table 5:** Univariate and multivariate analyses based on weighted activity-stratified logistic regression models.

	Model 1		Model 2		Model 3	
	OR (95%CI)	*p*-value	OR (95%CI)	*p*-value	OR (95%CI)	*p*-value
Underlying disease						
No						
SIRI	1.42(1.02 ~ 1.99)	0.039	1.25(0.83 ~ 1.89)	0.289	0.68(0.11 ~ 4.01)	0.667
SIRI (quartile)						
Q1 (<1.07)	Reference		Reference		Reference	
Q2 (1.07–1.52)	0.50(0.04 ~ 6.12)	0.588	0.54(0.04 ~ 8.37)	0.662	0.23(0.00 ~ 18.48)	0.510
Q3 (1.52–2.88)	1.15(0.14 ~ 9.38)	0.894	0.76(0.07 ~ 8.19)	0.823	0.38(0.00 ~ 55.74)	0.704
Q4 (≥2.88)	2.31(0.36 ~ 14.72)	0.376	1.04(0.12 ~ 9.10)	0.974	0.33(0.00 ~ 61.63)	0.676
*p* for trend	0.286		0.909		0.565	
Yes						
SIRI	1.56(1.27 ~ 1.90)	<0.001	1.57(1.26 ~ 1.95)	<0.001	2.11(1.44 ~ 3.08)	<0.001
SIRI (quartile)						
Q1 (<0.94)	Reference		Reference		Reference	
Q2 (0.94–1.35)	1.86 (0.55 ~ 6.27)	0.318	2.20(0.60 ~ 8.06)	0.236	1.50(0.29 ~ 7.89)	0.632
Q3 (1.35–2.65)	3.88 (1.24 ~ 12.11)	0.020	4.55 (1.38 ~ 15.06)	0.013	2.77 (0.56 ~ 13.78)	0.213
Q4 (≥2.65)	7.56 (2.47 ~ 23.12)	<0.001	8.12 (2.46 ~ 26.85)	<0.001	15.08(2.63 ~ 86.41)	0.002
*p* for trend	<0.001		<0.001		0.002	

Model 1: No adjustment. Model 2: adjusted for age, gender, BMI. Model 3: adjusted for age, gender, BMI, education level, smoking status, drinking; coronary heart disease, chronic obstructive pulmonary disease, diabetes, hypertension, TC, TG, LDL-C, HDL-C, L, M, N, PLT, HB, TP, Serum K^+^, Serum Cl^−^, Serum Ca^2+^, Serum Na^+^. CI, confidence interval; OR, ratio of ratios; SIRI, systemic inflammatory response index. The table shows that logistic regression showed that after correction for covariates, high SIRI was associated with an increased risk of PSE in the corrected model in the subgroup with underlying disease.

### 3.6 The linear relationship between SIRI and post-stroke epilepsy

**Figure 2:**
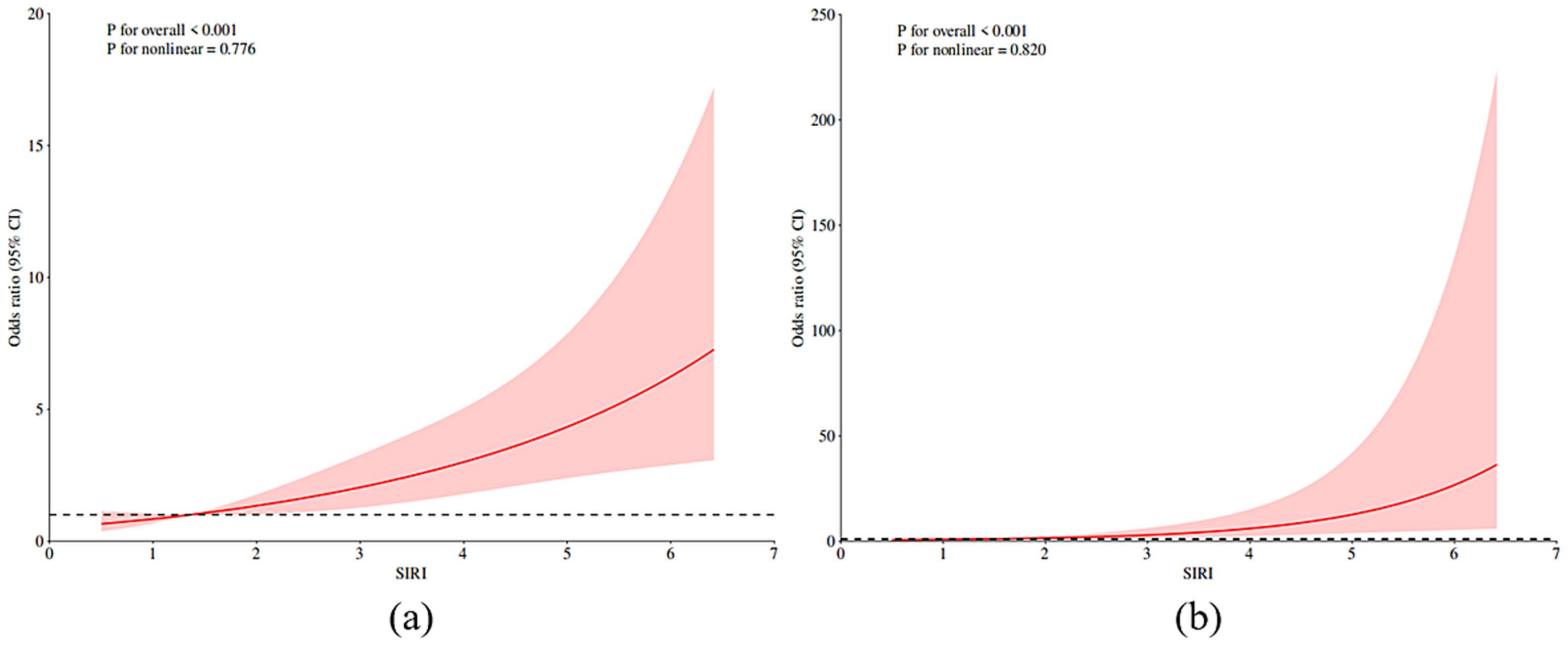
RCS prediction map. **(a)** RCS shows the nonlinear relationship between SIRI and post-stroke epilepsy. **(b)** RCS shows a nonlinear relationship between SIRI and post-stroke epilepsy after adjusting all covariates according to model 3. The fitting regression line is a red solid line; the black dotted line indicates the position where OR is equal to 1; shadowed area represents 95% CI. SIRI systemic inflammatory response index.

Without adjusting the covariates, we use RCS to better visualize the nonlinear relationship between SIRI and PSE (as shown in Figure 2a). The analysis based on Model 3 after adjusting all covariates shows that there is a significant threshold effect between SIRI and the probability of PSE, and the relationship is defined by a significant inflection point. The key findings show that when the SIRI level is lower than the inflection point, the incidence of PSE is relatively stable and low; however, once SIRI exceeds this inflection point, its probability of occurrence shows a sharp upward trend (as shown in Figure 2b). This result emphasizes that there may be a critical level of SIRI, beyond which the relevant pathophysiological processes will be significantly stimulated, thus greatly increasing the probability of epilepsy. The inflection point value identified in this study (1.36 in model 3) was calculated based on the current cohort data, but its specific value may be different due to population differences. Therefore, the universality and clinical application value of the inflection point need to be further verified by follow-up studies.

## 4 Discussion

Epilepsy is one of the common diseases after stroke, and its recurrent attacks can significantly increase the risk of disability and death. Based on the electronic medical record data of Dazhou Central Hospital from July 2021 to October 2024, this study used a retrospective analysis method to explore the association between SIRI and PSE. The following conclusions were drawn: The level of SIRI in patients with post-stroke epilepsy was significantly higher than that in patients without post-stroke epilepsy, and SIRI was positively correlated with PSE. After adjusting for multiple potential confounding factors, this correlation still exists. Through in-depth investigation of the results of restricted cubic spline analysis, we found that there is a significant nonlinear relationship between SIRI and PSE, and identified the inflection point of 1.36. The existence of this inflection point suggests that there may be a critical threshold for the effect of SIRI on the probability of PSE, which has important biological significance.

From the perspective of mechanism, this inflection point may reflect the critical state of post-stroke neuroinflammatory response from compensatory protection to pathological damage. When SIRI is lower than this threshold, the inflammatory response may still be in the controllable range and has not yet caused extensive neurovascular unit damage; when SIRI exceeds this inflection point, systemic inflammatory load may be sufficient to significantly destroy the integrity of the blood–brain barrier, activate glial cells, and promote the infiltration of a large number of inflammatory cells and the release of inflammatory factors, thereby significantly increasing the excitability of neural networks and the risk of epilepsy. This mechanism is consistent with our subsequent observation that in stroke patients with underlying diseases, the association between SIRI and PSE is more prominent, which may be due to the fact that these patients have a chronic inflammatory basis and are more likely to break through the inflammatory threshold, thus accelerating the occurrence of epilepsy. The above results further support the central role of immune inflammatory response in the occurrence of PSE. After stroke, the excessive infiltration of inflammatory cells such as neutrophils and monocytes and the imbalance of lymphocyte function can aggravate secondary brain injury by inducing oxidative stress, microcirculation failure, excitatory toxicity and other ways (16–20). SIRI is an indicator that comprehensively reflects the state of systemic inflammation, and its critical changes suggest that the occurrence of post-stroke epilepsy may be closely related to the loss of control of systemic inflammation. Therefore, monitoring SIRI levels may help early identification of high-risk patients and provide a theoretical basis for intervention strategies for inflammatory pathways.

In clinical practice, whole blood cell count (CBC) is widely used because of its convenience, instant results and economic advantages. Clinical studies have shown that inflammation-related indexes (such as SIRI) based on peripheral blood cell parameters have high stability and accessibility, and can be used as reliable indicators for systemic inflammation assessment. It is worth noting that the elevated level of SIRI is significantly positively correlated with the risk of hemorrhagic and ischemic stroke, and its predictive value has been verified in a number of cohort studies (21). Therefore, based on the SIRI calculated from neutrophils, lymphocytes, and monocytes, this indicator reflects the state of these three inflammatory cells and can more comprehensively assess the body ‘s inflammatory state and immune regulation balance (22). Evidence-based medical evidence shows that peripheral blood inflammatory cell ratio parameters (such as neutrophil/lymphocyte ratio NLR and platelet/lymphocyte ratio PLR) can be used as effective biomarkers to evaluate the progression and prognosis of acute ischemic stroke (AIS) (23–25). In addition, elevated SIRI levels are not only associated with the occurrence of stroke-associated pneumonia (SAP) (26), but also independently predict the risk of recurrence and all-cause death in patients receiving intravenous thrombolysis within 90 days (27). It is suggested that this index has important early warning value for poor clinical prognosis of AIS patients. The cohort study of Jin et al. (28) revealed that systemic immune inflammation index (SII) and SIRI had independent predictive value for stroke and all-cause mortality risk, and their correlation was not interfered by C-reactive protein level. Further analysis showed that elevated SII and SIRI levels could significantly increase the risk of ischemic and hemorrhagic stroke subtypes and overall mortality. Therefore, the systemic inflammatory response index, as a new and economical composite inflammatory index, combines three kinds of peripheral blood cells: neutrophils, lymphocytes and monocytes. At present, there are many studies on tumor and cardiovascular and cerebrovascular diseases, but there are few studies on post-stroke epilepsy and its correlation. Although the roles and functions of neutrophils, monocytes and lymphocytes play an important role in the pathophysiological mechanism of post-stroke epilepsy, the research on integrated inflammatory biomarkers in this field is still in its infancy, and its potential regulatory mechanism needs to be further explored. The purpose of this study was to explore the potential association between SIRI and PSE.

This study confirmed the link between SIRI and PSE, especially in stroke patients with underlying diseases, and found that elevated SIRI levels were associated with the occurrence of PSE. This suggests that effective control of underlying diseases may be beneficial for patients with post-stroke epilepsy. This also emphasizes the importance of the treatment and prevention of basic diseases. According to our findings, SIRI can be integrated into a wider range of stroke risk assessments, especially for high-risk populations. Routine screening may include monitoring SIRI levels and other identified risk factors. For individuals with elevated SIRI, interventions may involve lifestyle changes, such as monitoring and controlling blood pressure, blood glucose and blood lipids, improving lifestyle, quitting smoking and drinking, because these can help manage inflammation and oxidative stress, which is also a risk factor for post-stroke epilepsy.

In the analysis of inter-group differences, we found that there were statistically significant differences in total cholesterol, triglycerides, neutrophils, and SIRI (*p* < 0.001). The study of Zhuo et al. (29) confirmed that SIRI has significant clinical significance in the prognosis evaluation of patients with acute ischemic stroke (AIS). In this study, AIS patients with initial symptoms within 72 h were studied. Univariate regression analysis showed that SIRI level was significantly correlated with 90-day poor prognosis (mRS > 2) (OR = 1.561, 95% CI 1.308–1.863, *p* < 0.001), suggesting that it could be used as an independent predictor. In the multivariate logistic regression model, SIRI remained an independent prognostic value even after adjusting for demographic characteristics and multiple clinical risk factors (*p* < 0.05). In addition, the receiver operating characteristic curve (ROC) analysis showed that the predictive efficacy of SIRI for poor prognosis reached a moderate level (AUC: 0.714, 95% CI 0.658–0.765, *p* < 0.001). It is worth noting that when SIRI was combined with a number of conventional clinical indicators (including demographic parameters, basic disease history and biochemical indicators) to draw a joint ROC curve, it was found that SIRI had a significantly improved ability to identify poor prognosis of AIS (AUC: 0.829 vs. 0.790, *p* = 0.016). This finding suggests that the integration of SIRI indicators on the basis of the existing clinical evaluation system can effectively optimize the prognostic stratification management of AIS patients. But there are also literature conclusions to the contrary. Studies have suggested that (30), higher age is a protective factor for the recurrence of seizures after stroke. The mechanism may involve two factors. On the one hand, the sensitivity of the elderly to antiepileptic drugs is significantly enhanced due to physiological function degradation and pharmacokinetic changes. On the other hand, the widespread cortical atrophy and neuronal degeneration in elderly patients may lead to a decrease in the excitability of the central nervous system, thereby reducing the seizure threshold. It is worth noting that the current research on the correlation between age and the risk of recurrent seizures is still academically controversial, especially the conclusion that advanced age is a protective factor still needs to be verified by large sample prospective studies.

In the subgroup analysis, we found that the relationship between increased SIRI and epilepsy was more obvious in stroke patients with underlying diseases, and SIRI was positively correlated with the incidence of PSE. Through the interaction test, it was found that there was a significant interaction between SIRI and post-stroke epilepsy in people with underlying diseases (*p* < 0.001), suggesting that SIRI may be a reliable indicator for predicting the risk of post-stroke epilepsy. Logistic regression analysis showed that SIRI was positively correlated with PSE in the general population (OR: 1.49, 95% CI: 1.27–1.76, *p* < 0.001). In Model 3, after adjusting for all covariates, we found that SIRI was still significantly associated with the probability of PSE (OR: 1.99, 95% CI: 1.44–2.75, *p* < 0.001). Based on RCS, without adjusting covariates and adjusting all covariates, we observed that the prevalence of PSE increased with the increase of SIRI level. When SIRI exceeded 1.36, the incidence of PSE increased significantly. The above research results show that SIRI can help doctors effectively identify the risk of epilepsy after stroke, which is helpful for the prevention and management of stroke.

This study preliminarily revealed the potential correlation between SIRI and PSE, and provided an economical and high-quality evaluation strategy for clinical risk stratification. As an easy-to-obtain systemic inflammatory indicator, SIRI can be used to stratify the risk of epilepsy in stroke patients. Identifying high-risk post-stroke patients with elevated SIRI (especially beyond the inflection point value) will help clinicians to screen high-risk groups of PSE early and strengthen monitoring and management. It is worth noting that the incidence of subclinical epileptiform activity after stroke cannot be ignored. A study based on continuous electroencephalogram (cEEG) monitoring shows that the detection rate can reach 7% (95% CI: 3−12%) (31), and the proportion may be higher under the condition of cEEG continuous monitoring. This finding further emphasizes that for high-risk groups with elevated SIRI, it is of great clinical significance to actively use sensitive tools such as cEEG to capture epileptiform activity early, which provides the possibility for early intervention and improvement of prognosis. However, this study also has some limitations. Firstly, limited by the single-center retrospective study design, the sample size is relatively limited, and the data collection depends on the integrity of previous medical records. There may be information bias and residual confounding effects, which affect the robustness of the results. Secondly, the evaluation of the inflammatory index SIRI is only based on a single blood cell test data within 24 h of admission, and fails to include the time-series change characteristics of the indicators during hospitalization, which may cause measurement bias caused by the lack of dynamic information. In addition, although this study has included the history of diabetes as a covariate in the analysis, it did not include specific blood glucose level data. As a potential important confounding factor, acute blood glucose level may better reflect the influence of metabolic status on the occurrence of PSE than the history of diabetes. This limitation is mainly due to the lack of blood glucose data in some patients in retrospective studies. Future prospective studies should include a series of blood glucose monitoring data to more comprehensively assess the relationship between metabolic factors and post-stroke epilepsy. Finally, although as many covariates as possible are included, there are still unmeasured confounding factors that are not adjusted. In the future, it is necessary to further verify the mechanism and optimize clinical application value through a larger prospective cohort study combined with multi-omics data (such as inflammatory pathway analysis).

## 5 Conclusion

PSE has a high mortality and disability rate, which has a great impact on the quality of life of patients. Inflammatory response plays a key role in the occurrence, development and outcome of PSE. This study confirmed that there was a significant positive correlation between SIRI and PSE. The correlation between SIRI and PSE is more significant, especially in the stroke group with underlying diseases. Therefore, SIRI can be used as a clinical help for doctors to effectively identify the risk of epilepsy after stroke, and provide new ideas for the prevention and management of stroke. It should be pointed out that the causal relationship between SIRI and PSE cannot be established in this study, and a higher level of evidence-based basis needs to be obtained through prospective cohort studies. It is worth noting that as a new composite index that integrates multi-dimensional inflammatory indicators such as neutrophils, monocytes and lymphocytes, SIRI shows unique advantages in the evaluation of systemic inflammation. In view of its clinical application in PSE prediction, it is recommended to establish a unified prediction threshold standard through multi-center collaborative research. At the same time, it is necessary to carry out mechanism research from the molecular pathway and pathophysiological level, focusing on the dynamic regulation of inflammation-immune interaction network in disease progression.

## Data Availability

The datasets presented in this article are not readily available because this data is derived from patients treated at the hospital in recent years and is applicable to research related to stroke. Requests to access the datasets should be directed to 395394881@qq.com.
